# Patterns of sitting and mortality in the Nord-Trøndelag health study (HUNT)

**DOI:** 10.1186/s12966-016-0457-8

**Published:** 2017-01-26

**Authors:** Anne C. Grunseit, Josephine Y. Chau, Vegar Rangul, Turid Lingaas Holmen, Adrian Bauman

**Affiliations:** 1 Prevention Research Collaboration, School of Public Health, Charles Perkins Centre (D17), University of Sydney, New South Wales, 2006 Australia; 2 Department of Public health and General practice, HUNT Research Centre, Faculty of Medicine, NTNU – Norwegian University of Science and Technology, Levanger, Norway

**Keywords:** Sedentary behaviour, Epidemiology, Mortality, Cardiovascular disease

## Abstract

**Background:**

Current evidence concerning sedentary behaviour and mortality risk has used single time point assessments of sitting. Little is known about how changes in sitting levels over time affect subsequent mortality risk.

**Aim:**

To examine the associations between patterns of sitting time assessed at two time points 11 years apart and risk of all-cause and cardio-metabolic disease mortality.

**Methods:**

Participants were 25,651 adults aged > =20 years old from the Nord-Trøndelag Health Study with self-reported total sitting time in 1995-1997 (HUNT2) and 2006-2008 (HUNT3). Four categories characterised patterns of sitting: (1) low at HUNT2/ low at HUNT3, ‘consistently low sitting’; (2) low at HUNT2/high at HUNT3, ‘increased sitting’; (3) high at HUNT2/low at HUNT3, ‘reduced sitting’; and (4) high at HUNT2 /high at HUNT3, ‘consistently high sitting’. Associations of sitting pattern with all-cause and cardio-metabolic disease mortality were analysed using Cox regression adjusted for confounders.

**Results:**

Mean follow-up was 6.2 years (158880 person-years); 1212 participants died. Compared to ‘consistently low sitting’, adjusted hazard ratios for all-cause mortality were 1.51 (95% CI: 1.28–2.78), 1.03 (95% CI: 0.88–1.20), and 1.26 (95% CI: 1.06–1.51) for ‘increased sitting’, ‘reduced sitting’ and ‘consistently high sitting’ respectively.

**Conclusions:**

Examining patterns of sitting over time augments single time-point analyses of risk exposures associated with high sitting time. Whilst sitting habits can be stable over a long period, life events (e.g., changing jobs, retiring or illness) may influence sitting trajectories and therefore sitting-attributable risk. Reducing sitting may yield mortality risks comparable to a stable low-sitting pattern.

**Electronic supplementary material:**

The online version of this article (doi:10.1186/s12966-016-0457-8) contains supplementary material, which is available to authorized users.

## Introduction

Excessive sitting has demonstrated an association with adverse health outcomes, with higher amounts of sitting associated with higher risk of chronic diseases and all-cause mortality [1, 2]. Most studies on the association of prolonged sitting with all-cause mortality risk have involved single time point exposures of sitting, with only a few examining the risk associated with different patterns of sedentariness. León-Muñoz et al found in their group of older adults (aged 60+) significantly lower risk or all-cause mortality for those with consistently low sitting across assessments two years apart compared to those with consistently high sitting [3]. Another large study of post-menopausal women reported similar results although additionally found risk reductions among those who decreased their sitting between baseline and six years later not only for all-cause mortality but also cause-specific cardiovascular disease (CVD) and cancer mortality [4]. However, both these studies are confined to older adult populations.

Examining the effects of sitting using two or more exposure time points can demonstrate the associations between stable and changing ‘patterns of sitting’ and mortality outcomes. The current study examines different patterns of sitting at two time points 11 years apart and mortality approximately six years after the second measure in a population-based cohort in Norway.

## Methods

### Sampling

The Nord-Trøndelag Health Study (HUNT) is a large population-based cohort study conducted in the Nord-Trøndelag County located in central Norway (www.ntnu.no/hunt) [5]. Three health surveys have been conducted: HUNT1 (1984–1986), HUNT2 (1995–1997), and HUNT3 (2006–2008). The current analysis uses respondents from HUNT2 and HUNT3 who had self-reported sitting time for both surveys (*n =* 25651).

The study was approved by the Regional Committee for Medical and Health Research Ethics (REC Central) and the Norwegian Data Inspectorate and informed consent was obtained from all individual participants included in the study.

### Measures

#### Mortality

The study sample was linked to the Norwegian Causes of Death Registry by Statistics Norway, for the period from 1 January 1994 to 31 December 2013. The endpoints for this analysis were mortality from all causes and from cardio-metabolic diseases (CMD). Causes of death were coded based on the International Classification of Disease (ICD) and we identified deaths from CMD (diseases of the circulatory system, and endocrine, nutritional and metabolic diseases) using ICD-9 (codes 240–279, 390–459) for deaths occurring up to 1996 and ICD-10 (codes E10-E16, E65-E68, I00-I99) for deaths from 1996 onwards.

#### Sitting

Sitting was assessed with a single question ‘how many hours do you usually spend sitting down during a 24-h period?’ at both time points. Participants were prompted to recall sitting at work, mealtimes, watching television, sitting in a car, etc. We classified sitting eight hours or more as ‘high sitting’, based on a recent meta-analysis on sitting time and all-cause mortality [2]. Sitting pattern combined high (≥8 h/day) and low (<8 h/day) over the two surveys sitting yielding four categories (1) low at HUNT2/ low at HUNT3, ‘consistently low sitting’; (2) low at HUNT2/high at HUNT3, ‘increased sitting’; (3) high at HUNT2/low at HUNT3, ‘reduced sitting’; and (4) high at HUNT2 /high at HUNT3, ‘consistently high sitting’.

#### Covariates

Participants reported separately their time spent in ‘light’ physical activity and in ‘hard’ physical activity (average of hours/week in the last year: None, Less than 1 h, 1-2 h, 3 h or more) [6]. The ‘hard’ physical activity measure has acceptable measurement properties but the ‘light’ activity measure shows poor repeatability and validity [7]. Highest level of education [8], smoking status, and general health status were self-reported [9]. Diagnosis of a CMD was derived from questions used in the cardiovascular disease prevention program in Norway currently conducted by the Norwegian Institute of Public Health (https://www.fhi.no/). Body mass index (BMI) was calculated as weight/height^2^ (kg/m^2^) with underweight defined as BMI < 18.5 kg/m^2^, normal weight as BMI 18.5-24.9 kg/m^2^, overweight as BMI 25-29.9 kg/m^2^ and obese as ≥30 kg/m^2^.

### Statistical analysis

The relationship between sitting and mortality were analysed using Cox proportional hazards models for both all-cause and CMD mortality. Separate models were run using the single time point dichotomous sitting exposure variables measured at HUNT2 and HUNT3 (reference group sitting <8 h/day), and sitting pattern from HUNT2/HUNT3 combined (reference group consistently low sitting). Age was the time scale used with age at screening at HUNT3 as the entry time and age at death/censoring as the exit time. For comparability between the single and multiple-point exposure variables, analyses were restricted to respondents with data for sitting at both time points, and the time-at-risk fixed from HUNT3 (as sitting pattern could only be generated for those who had survived to the HUNT3 assessment). All analyses were adjusted for sex, and HUNT2 measures of level of education, light and hard physical activity, smoking status, body mass index (BMI), general health and self-reported diagnosis of CMD disease. Competing risks models were used for CMD mortality to account for those dying of other causes [10]. Results are presented as adjusted hazard ratios (HR) for all-cause mortality and subhazard ratio (SHR) for CMD mortality, along with crude mortality rates for each level of the exposure variables. Sensitivity analyses were conducted excluding deaths within three years of HUNT3 as well as those diagnosed with CMD, in poor or ‘not so good’ health, smoking, or who were obese at HUNT2.

## Results

The sample characteristics for the 25651 participants who had complete data for the exposure variables and the covariates (the analytic sample) are shown in Table 1. Briefly, most were middle aged, had at least high/vocational schooling (73.3%), undertook light exercise for at least 1–2 h per week (68.4%), were not currently smoking (73.2%) and were overweight or obese (57.8%). Less than five percent reported having or ever had myocardial infarction, angina, stroke or diabetes (CMD). Thirty-seven percent were sitting more than eight hours at HUNT2, and just under a quarter (24.9%) at HUNT3. The majority (54.3%) reported consistently low levels of sitting, 16.4% reported consistently high sitting, 8.4% changed from low to high sitting, and 20.8% changed from high to low sitting from the HUNT2 to HUNT3 surveys.

**Table 1:** Demographic characteristics by sitting level at HUNT2, HUNT3 and across HUNT2/HUNT3 (sitting pattern) for the analytic sample (*n =* 25651)

	All	HUNT 2 sitting		HUNT 3 sitting		Sitting pattern H2/H3			
Characteristic at HUNT 2	*n =* 25651	<8 h (*n =* 16088)	≥8 h (*n =* 9563)	<8 h (*n =* 19275)	≥ 8 h (*n =* 6376)	Low/Low (*n =* 13930)	Low/High (*n =* 2158)	High/Low (*n =* 5345)	High/High (*n =* 4218)
Age*	n col %	n col %	n col %	n col %	n col %	n col %	n col %	n col %	n col %
19-35 years	6,120	3,872	2,248	4,566	1,554	3,315	557	1,251	997
	23.9	24.1	23.5	23.7	24.4	23.8	25.8	23.4	23.7
>35-50 years	10,530	6,263	4,267	7,542	2,988	5,433	830	2,109	2,158
	41.1	38.9	44.6	39.1	46.9	39.0	38.5	39.5	51.2
>50-65 years	7,030	4,506	2,524	5,645	1,385	3,991	515	1,654	870
	27.4	28.0	26.4	29.3	21.7	28.7	23.9	30.9	20.6
>65 years	1,966	1,444	522	1,519	447	1,188	256	331	191
	7.7	9.0	5.5	7.9	7.0	8.5	11.9	6.2	4.5
Gender									
Female	13,497	9,050	4,447	10,518	2,979	7,967	1,083	2,551	1,896
	52.6	56.3	46.5	54.6	46.7	57.2	50.2	47.7	45.0
Male	12,154	7,038	5,116	8,757	3,397	5,963	1,075	2,794	2,322
	47.4	43.8	53.5	45.4	53.3	42.8	49.8	52.3	55.1
Education									
Primary school	6,521	4,831	1,690	5,458	1,063	4,285	546	1,173	517
	25.4	30.0	17.7	28.3	16.7	30.8	25.3	22.0	12.3
High school vocational school	9,752	6,467	3,285	7,643	2,109	5,687	780	1,956	1,329
	38.0	40.2	34.4	39.7	33.1	40.8	36.1	36.6	31.5
University qualifying exams	2,587	1,414	1,173	1,788	799	1,175	239	613	560
	10.1	8.8	12.3	9.3	12.5	8.4	11.1	11.5	13.3
University (<4 years)	3,920	1,940	1,980	2,543	1,377	1,605	335	938	1,042
	15.3	12.1	20.7	13.2	21.6	11.5	15.5	17.6	24.7
University (≥4 years)	2,545	1,208	1,337	1,583	962	987	221	596	741
	9.9	7.5	14.0	8.2	15.1	7.1	10.2	11.2	17.6
Education unknown	326	228	98	260	66	191	37	69	29
	1.3	1.4	1.0	1.4	1.0	1.4	1.7	1.3	0.7
Light exercise									
Light exercise - none	1,306	833	473	966	340	711	122	255	218
	5.1	5.2	5.0	5.0	5.3	5.1	5.7	4.8	5.2
<1 h/week	4,149	2,487	1,662	3,056	1,093	2,128	359	928	734
	16.2	15.5	17.4	15.9	17.1	15.3	16.6	17.4	17.4
1–2 h/week	9,211	5,632	3,579	6,892	2,319	4,919	713	1,973	1,606
	35.9	35.0	37.4	35.8	36.4	35.3	33.0	36.9	38.1
3+ hours/week	8,335	5,338	2,997	6,279	2,056	4,588	750	1,691	1,306
	32.5	33.2	31.3	32.6	32.3	32.9	34.8	31.6	31.0
Light exercise unknown	2,650	1,798	852	2,082	568	1,584	214	498	354
	10.3	11.2	8.9	10.8	8.9	11.4	9.9	9.3	8.4
Hard exercise									
Hard exercise—none	6,599	4,121	2,478	4,960	1,639	3,538	583	1,422	1,056
	25.7	25.6	25.9	25.7	25.7	25.4	27.0	26.6	25.0
<1 h/week	5,943	3,477	2,466	4,292	1,651	2,990	487	1,302	1,164
	23.2	21.6	25.8	22.3	25.9	21.5	22.6	24.4	27.6
1–2 h/week	5,163	3,082	2,081	3,758	1,405	2,661	421	1,097	984
	20.1	19.2	21.8	19.5	22.0	19.1	19.5	20.5	23.3
3+ hours/week	2,547	1,605	942	1,933	614	1,385	220	548	394
	9.9	10.0	9.9	10.0	9.6	9.9	10.2	10.3	9.3
Hard exercise unknown	5,399	3,803	1,596	4,332	1,067	3,356	447	976	620
	21.1	23.6	16.7	22.5	16.7	24.1	20.7	18.3	14.7
Smoking									
Never smoked	11,691	7,352	4,339	8,811	2,880	6,457	895	2,354	1,985
	45.6	45.7	45.4	45.7	45.2	46.4	41.5	44.0	47.1
Ex-smoker	6,433	3,935	2,498	4,772	1,661	3,367	568	1,405	1,093
	25.1	24.5	26.1	24.8	26.1	24.2	26.3	26.3	25.9
Current smoker	6,881	4,359	2,522	5,185	1,696	3,718	641	1,467	1,055
	26.8	27.1	26.4	26.9	26.6	26.7	29.7	27.5	25.0
Smoking unknown	646	442	204	507	139	388	54	119	85
	2.5	2.8	2.1	2.6	2.2	2.8	2.5	2.2	2.0
BMI									
BMI < 18.5	140	92	48	103	37	80	12	23	25
	0.6	0.6	0.5	0.5	0.6	0.6	0.6	0.4	0.6
BMI 18.5–24.9	10,646	6,708	3,938	8,049	2,597	5,868	840	2,181	1,757
	41.5	41.7	41.2	41.8	40.7	42.1	38.9	40.8	41.7
BMI 25- < 30	11,402	7,119	4,283	8,593	2,809	6,165	954	2,428	1,855
	44.5	44.3	44.8	44.6	44.1	44.3	44.2	45.4	44.0
30+	3,412	2,145	1,267	2,498	914	1,796	349	702	565
	13.3	13.3	13.3	13.0	14.3	12.9	16.2	13.1	13.4
BMI unknown	51	24	27	32	19	21	3	11	16
	0.2	0.2	0.3	0.2	0.3	0.2	0.1	0.2	0.4
General health									
Poor	204	124	80	145	59	99	25	46	34
	0.8	0.8	0.8	0.8	0.9	0.7	1.2	0.9	0.8
Not so good	4,785	3,154	1,631	3,651	1,134	2,681	473	970	661
	18.7	19.6	17.1	18.9	17.8	19.3	21.9	18.2	15.7
Good	15,491	9,661	5,830	11,657	3,834	8,394	1,267	3,263	2,567
	60.4	60.1	61.0	60.5	60.1	60.3	58.7	61.1	60.9
Very good	5,004	3,046	1,958	3,702	1,302	2,667	379	1,035	923
	19.5	18.9	20.5	19.2	20.4	19.2	17.6	19.4	21.9
General health unknown	167	103	64	120	47	89	14	31	33
	0.7	0.6	0.7	0.6	0.7	0.6	0.7	0.6	0.8
CMD									
No CMD & cancer	23,925	14,897	9,028	17,966	5,959	12,930	1,967	5,036	3,992
	93.3	92.6	94.4	93.2	93.5	92.8	91.2	94.2	94.6
CMD	1,172	768	404	852	320	623	145	229	175
	4.6	4.8	4.2	4.4	5.0	4.5	6.7	4.3	4.2
CMD unknown	554	423	131	457	97	377	46	80	51
	2.2	2.6	1.4	2.4	1.5	2.7	2.1	1.5	1.2

* Five respondents with a valid age at HUNT 3 (and were therefore included in the analytic sample) did not have an age at HUNT 2 screening, hence percentages are calculated on *n*=25646

Among the analytic sample, 1212 died during the (average) 6.19 year follow-up post HUNT3 (158880 person years). The crude all-cause death rate for those sitting eight hours or more at HUNT2 was 6.7 per 1000 person-years and 2.4 deaths per 1000 person-years for CMD mortality (Additional file 1: Table S1). After adjusting for covariates, there was no greater risk of all-cause or CMD-specific death for those sitting more (compared with less) than eight hours (HR 1.02 (95% CI: 0.91–1.16), and SHR 1.20 (95% CI: 0.96–1.49) respectively. For those with high sitting time at HUNT3, risk of death from all causes and CMD were significantly higher (HR 1.37 (95% CI: 1.21–1.56), and SHR 1.58 (95% CI: 1.28–1.97) respectively (Additional file 1: Table S1). Similarly, when sitting was operationalised as continuous (hours/day) there was with no significant association for HUNT2 but a 5% per sitting-hour and 6% per sitting-hour increased hazard at HUNT3 for all-cause and CMD mortality respectively (data not shown).

When sitting patterns were analysed, compared to people with ‘consistently low sitting’, the highest risk was observed among those who had ‘increased sitting’ both in terms of crude and adjusted rates and for all-cause and CMD-specific mortality (Table 2). Specifically, the adjusted hazard of dying increased by 50% for all-cause mortality and 85% for CMD mortality among participants with low sitting time at HUNT2 and high sitting time at HUNT3. These rates were higher than the corresponding increased hazards estimated for participants who reported ‘consistently high sitting’ (25% and 49% respectively). Those who had ‘reduced sitting’ were not significantly different in the risk of dying to those who reported ‘consistently low sitting’. The population attributable fractions indicating the proportion of deaths saved if the whole sample were to have consistently low sitting were 8.8% (95% CI: 3.8%-13.1%) and 13.9% (95% CI: 6.7%-20.6%) for all-cause and CMD-specific mortality respectively.

**Table 2:** Crude death rates per 1000 person years (PY) and adjusted hazard/subhazard ratios for all-cause and cause-specific mortality across multiple-point sitting variables in HUNT 2 and HUNT 3

Two time-point analyses						
Consistently low sitting	Increased sitting		Reduced sitting		Consistently high sitting	
Low sitting H2 & H3 (ref cat)	Low sitting H2/high sitting H3		High sitting H2/Low sitting H3		High sitting H2 & H3	
Crude rate /1000PY (unexposed)	Crude rate /1000PY (exposed)	Adj HR/SHR (95% CI)	Crude rate /1000PY (exposed)	Adj HR/SHR (95%CI)	Crude rate /1000PY (exposed)	Adj HR/SHR (95% CI)
7.21	14.49	1.50 (1.27–1.77)	6.84	1.03 (0.88–1.20)	6.58	1.25 (1.05–1.49)
2.03	5.44	1.85 (1.38–2.48)	2.44	1.29 (0.98–1.69)	2.30	1.49 (1.11–2.01)

Sensitivity analyses for sitting pattern (Additional file 2: Table S2) excluding those dying within up to three years of HUNT3 showed little change in effect size and statistical significance from the full-sample analysis except the all-cause mortality HR for consistently high sitting became non-significant at two years, and became significant for reduced sitting for CMD-specific mortality (HR = 1.33 > dying 1 year post-HUNT3, HR = 1.37 > dying 2 years post-HUNT3). Similarly, excluding respondents with CMD or poor/not so good health or who were smokers at HUNT2 also attenuated the effect of high sitting for all-cause and CMD-specific mortality perhaps in part due to reduced power with the smaller sample. However reduced sitting became significant for CMD-specific mortality when those with CMD or poor health at HUNT2 were excluded (Additional file 2: Table S2).

## Discussion

Our results extend previous epidemiological studies with only one baseline exposure measurement of sitting time and those investigating cumulative effects of excessive sitting and impact of changes in sitting over time in older populations [3, 4]. In brief, our analysis demonstrated that proximal measures show a stronger relationship, but a behavioural pattern can demonstrate the impact of changes in sitting behaviour as well as sustained high and low sitting. Further, targeting of interventions to reduce sedentary behaviour can be informed by these data as those reducing their sitting time show a reduction in mortality risk. Finally, analytic strategies which operationalise change across time may reveal more nuanced relationships between sedentary behaviour and mortality. Thus, understanding the risks associated with patterns of sitting have implications for measurement, interventions and future analytic strategies of sitting behaviour and health outcomes.

Overall, most adults in this large population sample reported sitting less than eight hours per day consistent with previous research involving Scandinavian samples [11]. Over 70% reported a stable sitting pattern 11 years apart. As with prior research [12], the effect of sitting time was stronger for CMD-related than all-cause mortality. Between the two single time-point analyses, significant effects were only seen with the more proximal exposure measure (HUNT3), as the behaviour-outcome association is stronger with more recent measures as behaviour may change over time.

The mortality risk for those who reported high levels of sitting at HUNT3 was significantly higher irrespective of whether they reported low or high levels of sitting at HUNT2. Those who sat less than eight hours/day at HUNT3 despite reporting high sitting at HUNT2 showed comparable risk of dying as those reporting ‘consistently low sitting’ levels, reminiscent of findings reported previously demonstrating that change towards healthier practices over time such as uptake of physical activity and smoking cessation reduces mortality risk [13, 14]. By contrast, León-Muñoz et al [3] found similar and non-significant all-cause mortality risk for those who reduced their sitting as those who increased their sitting (compared with consistently high sitting), concluding that the relevant exposure is cumulative [3]. Our analysis however, had a longer inter-assessment interval (11 years vs 2 years) and had a larger (>26000 vs 2625) more diverse (in terms of age) sample size which may have made it more sensitive to intergroup variation. Further, our findings are consistent with those of the larger study, albeit based on post-menopausal women only, by Lee et al [4].

Change between surveys most commonly occurred from sitting more than eight hours at HUNT2 but not at HUNT3. Although similar (within 2%) to the reference group in age, physical activity (where known), smoking, BMI, general health and reporting CMD (at HUNT2), there were more males and they were better educated. Further investigation showed that of the 71% in this group who reported working (HUNT3), 23% moved from a mainly sedentary job to a non-sedentary occupation (involving much walking, lifting or physical labour) between surveys. Therefore, given the high contribution of occupational sitting to total sitting [15] this group’s pattern may reflect a change in occupation; a change which meant their mortality risk was comparable to a consistently low sitting pattern. However, the sensitivity analyses for CMD-specific mortality, but not all-cause, suggest that this group may still carry some residual risk, especially among the otherwise healthy in this group.

Participants reporting high sitting at HUNT3 but not HUNT2 represented the most at-risk group. Although their health risk profile was similar to those reporting stable low sitting levels, they had the highest proportion aged over 65-year age group (11.9% vs 7.7% overall) and almost 45% had moved from an active to sedentary occupation. Supplementary analyses showed this group was more likely to have developed CMD between surveys and report reduced self-rated health at HUNT3 despite reporting good/very good health at HUNT2; therefore the increase in sitting could feasibly reflect newly acquired health problems, raising the question of reverse causality [16]. However, because we have no detailed information on the sequencing of sitting and the onset of poorer health, we cannot disentangle these factors. Including HUNT3 measures of smoking and physical activity did not appreciably change effect sizes or statistical significance (Additional file 3: Table S3).

Finally, the second highest mortality risk was among those with high levels of sitting at both time points. Over half of this group were aged 30-50 years (vs 41% overall), were highly educated, majority male and had good general health ratings. Three-quarters of those working at HUNT3 had sedentary occupations, and one-fifth were in a management position (vs 3% in the stable low group). Taken together, this group appears to be drawn from a higher occupational status population, as physically active as the other groups, but sedentary at work. Although not the most at-risk group, they show elevated mortality risk and perhaps with age may be at greater risk if they maintain current behavioural patterns; their risk profile but accessibility through the workplace makes this group suitable target for intervention.

Strengths of this analysis include the large prospective, population-based sample, identical measures for the exposure variable at both time points and the inclusion of a wide range of potential confounders. Further, our threshold for high sitting was based on a previous meta-analysis [2]. Study findings may be limited by the restriction to those who survived to the second survey hence representing a healthier group in general. The missing data rates for the two physical activity measures and their variability across the different levels of the exposure variables may mean that the full effect of physical activity, especially for vigorous physical activity, may not have been estimated. However, including a missing category allowed us to estimate the association with mortality and retain these participants’ sitting data in the analysis.

In conclusion, our analysis provides evidence that sitting habits can show long-term stability, but subgroups, such as those changing or leaving jobs or developing illness may have variable sitting trajectories and different risks attributable to the patterns of sitting. Investigations of patterns of sitting add to previous single time-point analyses and may reflect a more realistic operationalization of exposure to excessive sitting especially among groups who are experiencing change in their health or lifestyle. Future analyses could examine the joint effects with physical activity as reported previously with single time-point analyses [17] and explore further the potentially divergent effects with otherwise healthy and unhealthy subpopulations.

## Additional files

Additional file 1: Table S1. Crude death rates per 1000 person years (PY) and adjusted hazard/subhazard ratios for all-cause and cause-specific mortality across single-point sitting variables in HUNT 2 and HUNT 3 (DOC 30 kb)
Additional file 2: Table S2. Sensitivity analyses* for sitting patterns and mortality risk (DOC 49 kb)
Additional file 3: Table S3. Sensitivity analyses adding HUNT3 smoking and physical activity measures. (DOC 31 kb)

## Abbreviations

BMI: Body mass index
CMD: Cardiometabolic disease
CVD: Cardiovascular disease
HR: Hazard ratio
HUNT: Nord-Trøndelag Health Study
ICD: Classification of Disease
PY: Person years
SHR: Subhazard ratio
