# Supplementary material for: Patterns of sitting and mortality in the Nord-Trøndelag health study (HUNT)
Source: Int J Behav Nutr Phys Act. 2017 Jan 26;14:8. doi: 10.1186/s12966-016-0457-8 (PMC5267382; doi:10.1186/s12966-016-0457-8)
Supplement: Additional file 1: Table S1. — Crude death rates per 1000 person years (PY) and adjusted hazard/subhazard ratios for all-cause and cause-specific mortality across single-point sitting variables in HUNT 2 and HUNT 3 (DOC 30 kb) [file 12966_2016_457_MOESM1_ESM.doc]

Supplementary Table S1. Crude death rates per 1000 person years (PY) and adjusted hazard/subhazard ratios for all-cause and cause-specific mortality across single-point sitting variables in HUNT 2 and HUNT 3

| **Single time point analyses** | | | | | | | |
| --- | --- | --- | --- | --- | --- | --- | --- |
|  | **Deaths** | **Sitting ≤8 hours H2** | **Sitting ≥8 hours H2** | | **Sitting ≤8 hours H3** | **Sitting ≥8 hours H3** | |
|  |  | **Crude rate /1000PY (unexposed)** | **Crude rate /1000PY (exposed)** | **Adj HR/SHR (95%CI)** | **Crude rate /1000PY (unexposed)** | **Crude rate /1000PY (exposed)** | **Adj HR/SHR (95%CI)** |
| **All-cause mortality** | 1212 | 8.16 | 6.73 | 1.02  (0.90-1.16) | 7.10 | 9.22 | 1.36  (1.20-1.55) |
| **CVD-metab mortality** | 388 | 2.48 | 2.38 | 1.18  (0.95-1.47) | 3.35 | 3.35 | 1.54  (1.24-1.92) |
