# Supplementary material for: Patterns of sitting and mortality in the Nord-Trøndelag health study (HUNT)
Source: Int J Behav Nutr Phys Act. 2017 Jan 26;14:8. doi: 10.1186/s12966-016-0457-8 (PMC5267382; doi:10.1186/s12966-016-0457-8)
Supplement: Additional file 2: Table S2. — Sensitivity analyses* for sitting patterns and mortality risk (DOC 49 kb) [file 12966_2016_457_MOESM2_ESM.doc]

Supplementary Table S2: Sensitivity analyses* for sitting patterns and mortality risk

|  | **n died** | **Low/High** | **High/Low** | **High/High** |
| --- | --- | --- | --- | --- |
| **All-cause mortality Main analysis** | 1212 | **1.5** | **1.03** | **1.25** |
|  |  | **(1.27-1.77)** | **(0.88-1.20)** | **(1.05-1.49)** |
| **Exclude died <1 year post HUNT 3** | 1094 | 1.42 | 1.04 | 1.21 |
|  |  | (1.19-1.70) | (.89-1.23) | (1.01-1.46) |
| **Exclude died <2 years post HUNT 3** | 926 | 1.32 | 1.06 | 1.17 |
|  |  | (1.09 -1.61) | (.89-1.26) | (.95-1.43) |
| **Exclude died <3 years post HUNT 3** | 747 | 1.23 | 1.11 | 1.14 |
|  |  | (.99-1.54) | (.92-1.34) | ( .90-1.43) |
| **exclude CMD at HUNT2** | 969 | 1.64 | 1.08 | 1.22 |
|  |  | (1.36-1.97) | (.92-1.29) | (1.00-1.49) |
| **excludes Poor/not so good at HUNT2** | 803 | 1.62 | 1.04 | 1.20 |
|  |  | (1.32-1.98) | (.86-1.25) | (.96-1.50) |
| **excludes current smokers at HUNT2** | 805 | 1.45 | 0.92 | 1.16 |
|  |  | (1.19-1.78) | (.76-1.11) | ( .93-1.44) |
| **excludes overweight/obese H2** | 982 | 1.59 | 1.05 | 1.22 |
|  |  | (1.32-1.91) | (.88-1.25) | (1.00-1.48) |
| **CVD-metab mortality Main analysis** | 388 | **1.85** | **1.29** | **1.49** |
|  |  | **(1.38-2.48)** | **(0.98-1.69)** | **(1.11-2.01)** |
| **Exclude died <1 year post HUNT 3** | 346 | 1.83 | 1.33 | 1.47 |
|  |  | (1.34-2.49) | (1.00-1.78) | (1.08-2.01) |
| **Exclude died <2 years post HUNT 3** | 289 | 1.76 | 1.37 | 1.44 |
|  |  | (1.26-2.48) | (1.01-1.87) | (1.02-2.03) |
| **Exclude died <3 years post HUNT 3** | 230 | 1.74 | 1.36 | 1.49 |
|  |  | (1.19-2.55) | (.96-1.91) | (1.01-2.20) |
| **exclude CMD at HUNT2** | 257 | 2.11 | 1.44 | 1.20 |
|  |  | (1.49-2.99) | (1.04-1.98) | (0.79-1.82) |
| **excludes poor/not so good at HUNT2** | 255 | 2.05 | 1.48 | 1.39 |
|  |  | (1.43-2.94) | (1.07-2.06) | (.92-2.09) |
| **excludes current smokers at HUNT2** | 273 | 1.63 | 1.02 | 1.35 |
|  |  | (1.14 -2.32) | (.73-1.44) | (.95-1.91) |
| **excludes overweight/obese at HUNT2** | 304 | 1.98 | 1.36 | 1.51 |
|  |  | (1.42-2.76) | (1.00-1.86) | (1.08-2.13) |

* Excluding respondents dying up to three years post-HUNT3 and excluding unhealthy subpopulations at HUNT2
