# Supplementary material for: Patterns of sitting and mortality in the Nord-Trøndelag health study (HUNT)
Source: Int J Behav Nutr Phys Act. 2017 Jan 26;14:8. doi: 10.1186/s12966-016-0457-8 (PMC5267382; doi:10.1186/s12966-016-0457-8)
Supplement: Additional file 3: Table S3. — Sensitivity analyses adding HUNT3 smoking and physical activity measures. (DOC 31 kb) [file 12966_2016_457_MOESM3_ESM.doc]

Supplementary Table S3: Sensitivity analyses adding HUNT3 smoking and physical activity measures

|  |  | Two time-point | | |
| --- | --- | --- | --- | --- |
|  | n died | **Low/High** | **High/Low** | **High/High** |
|  |  |  |  |  |
| **All-cause mortality Main analysis** | 1212 | **1.5** | **1.03** | **1.25** |
|  |  | **(1.27-1.77)** | **(0.88-1.20)** | **(1.05-1.49)** |
| Add HUNT 3 smoking status, light and hard PA | 1212 | 1.43 | 1.04 | 1.21 |
|  |  | (1.21-1.69) | (.89-1.21) | (1.02-1.45) |
| **CVD-metab mortality Main analysis** | 388 | **1.85** | **1.29** | **1.49** |
|  |  | **(1.38-2.48)** | **(0.98-1.69)** | **(1.11-2.01)** |
| Add HUNT 3 smoking status, light and hard PA | 388 | 1.71 | 1.32 | 1.42 |
|  |  | (1.27-2.30) | (1.00-1.74) | (1.05-1.92) |
